# MicroED: Unveiling the Structural Chemistry of Plant Biomineralisation

**DOI:** 10.3390/molecules29204916

**Published:** 2024-10-17

**Authors:** Damian Trzybiński, Marcin Ziemniak, Barbara Olech, Szymon Sutuła, Tomasz Góral, Olga Bemowska-Kałabun, Krzysztof Brzost, Małgorzata Wierzbicka, Krzysztof Woźniak

**Affiliations:** 1 Faculty of Chemistry, Biological and Chemical Research Centre, University of Warsaw, Żwirki i Wigury 101, 02-089 Warsaw, Poland; dtrzybinski@cnbc.uw.edu.pl (D.T.); mziemniak@chem.uw.edu.pl (M.Z.); b.gruza@uw.edu.pl (B.O.); s.sutula@uw.edu.pl (S.S.); 2 Centre of New Technologies, University of Warsaw, Banacha 2C, 02-097 Warsaw, Poland; t.goral@cent.uw.edu.pl; 3 Isotope Laboratory, Faculty’s Independent Centres, Faculty of Biology, University of Warsaw, Miecznikowa 1, 02-096 Warsaw, Poland; o.bemowska@uw.edu.pl; 4 Department of Ecotoxicology, Institute of Environmental Biology, Faculty of Biology, University of Warsaw, Miecznikowa 1, 02-096 Warsaw, Poland; brzost@biol.uw.edu.pl

**Keywords:** electron diffraction, microED, plant crystals, Armeria maritima, crystallography, bio-induced crystallisation

## Abstract

Plants are able to produce various types of crystals through metabolic processes, serving functions ranging from herbivore deterrence to photosynthetic efficiency. However, the structural analysis of these crystals has remained challenging due to their small and often imperfect nature, which renders traditional X-ray diffraction techniques unsuitable. This study explores the use of Microcrystal Electron Diffraction (microED) as a novel method for the structural analysis of plant-derived microcrystals, focusing on *Armeria maritima* (Milld.), a halophytic plant known for its biomineralisation capabilities. In this study, *A. maritima* plants were cultivated under controlled laboratory conditions with exposure to cadmium and thallium to induce the formation of crystalline deposits on their leaf surfaces. These deposits were analysed using microED, revealing the presence of sodium chloride (halite), sodium sulphate (thénardite), and calcium sulphate dihydrate (gypsum). Our findings highlight the potential of microED as a versatile tool in plant science, capable of providing detailed structural insights into biomineralisation processes, even from minimal and imperfect crystalline samples. The application of microED in this context not only advances the present understanding of *A. maritima*’s adaptation to saline environments but also opens new avenues for exploring the structural chemistry of biomineralisation in other plant species. Our study advocates for the broader adoption of microED in botanical research, especially when dealing with challenging crystallographic problems.

## 1. Introduction

It is widely acknowledged that plants play an essential role in the existence of nearly all known ecosystems on Earth, contributing to them in numerous important ways. Among their roles, they are responsible for oxygen production, releasing significant amounts of water into the atmosphere, providing nutrients to most terrestrial organisms, and participating in vital geochemical cycles [1,2,3]. Despite extensive scientific research on plant biochemistry and physiology, certain aspects remain elusive. One of them is biomineralisation—an ability to produce crystals by a living organism—which has been observed in many plant species [4,5]. Typically, these crystals are products of metabolic processes and are deposited in either vacuoles or the cytoplasm [6,7]. In some instances, the crystals are located in specialised cells such as idioblasts [8,9] that exist on the leaf surface [10,11]. These crystals can exhibit various morphologies [12,13], often distinctive to individual plant species or broader taxonomic groups, making them potentially useful in plant identification [14,15,16]. Among the most prevalent types of crystals are those composed of calcium salts, notably calcium oxalate and calcium carbonate [6,8]. On rarer occasions, other inorganic compounds may also form such structures [8,17,18]. Plant crystals have diverse functions, including defending against herbivores, maintaining calcium homeostasis, regulating other ionic equilibria, providing mechanical support, and aiding in photosynthesis by capturing and focusing incoming sunlight [6,8,19,20,21,22].

Research on plant-origin crystalline materials primarily focuses on their elemental composition and morphological description [8,13]. While the importance of these studies is evident, a comprehensive understanding of these materials’ production and function within the plant kingdom remains ambiguous [5,23]. Many studies in this field tend to overlook the short- and long-range structural order of ionic or molecular constituents of the crystals. It should also be taken into account that chemical compounds can display polymorphism, a phenomenon where multiple crystalline phases of a single compound exist due to varying spatial arrangements of its components [24]. The structural complexity increases further when considering the hydration of these systems, which can lead to different possible numbers of water molecules in separate crystalline forms [25]. Diffraction methods provide insights into these structural nuances [26,27,28]. However, a challenge arises since plant-derived samples often contain only a limited amount of crystalline material, and these crystals tend to be exceptionally small, making them unsuitable for X-ray diffraction. A potential solution is electron diffraction (ED), which has recently emerged as a powerful tool in structural chemistry and biochemistry [29,30,31,32,33]. A key benefit of this method is the strong interaction between condensed matter and electrons, enabling the acquisition of diffraction data even from minuscule crystals [29]. Furthermore, this technique also offers other advantages, including a pronounced difference in atomic scattering factors between ions and neutral atoms [30]. This unique feature permits the detection of atomic oxidation states, particularly in low-resolution data, which can be advantageous for analysing defective or contaminated inorganic samples [30,34].

Among the various ED methods, Microcrystal Electron Diffraction (microED) is the most versatile, allowing for crystal structure determination even for samples smaller than 1 μm^3^. Importantly, the data collection is fast, enabling the screening of numerous crystals in a relatively short time [35,36]. Moreover, the processing of diffraction patterns mostly aligns with techniques used in classic X-ray crystallography [37]. Historically, ED was a tool for a niche group of specialised researchers. However, with the advent of tailored equipment and software, it has become more intuitive and accessible to the broader scientific community [38]. Consequently, applications of microED have expanded to diverse domains, including materials science [29], pharmaceutical research [32], and biochemistry [33,39]. Recently, ED has also been applied to the study of biological structures, namely biogenic guanidine crystals extracted from animal tissues [40].

**Figure 1:**
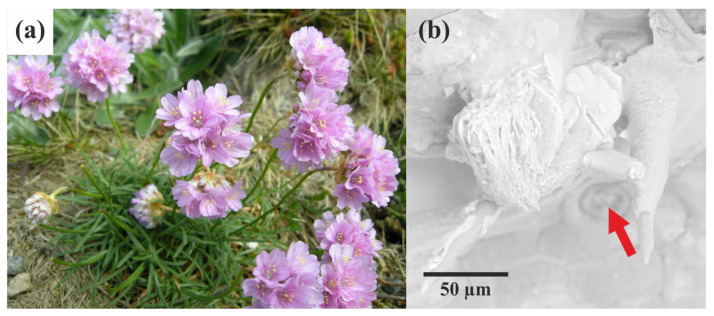
*Armeria maritima* is depicted as follows: (**a**) a general view of the plant in the flowering phase. This perennial herbaceous plant is characterised by its narrow lanceolate leaves arranged in a rosette and its purple capitate inflorescences (photo: Arnstein Rønning); (**b**) SEM image showing the salt gland (marked by red arrow) and the polycrystalline material excreted by the gland.

The aim of this short article is to showcase the potential of microED in the structural analysis of plant-derived crystals, focusing on its ability to resolve challenges associated with small crystal sizes. *Armeria maritima* (Milld.) is a wild plant (Figure 1a) known for producing crystalline material in a fascinating yet not fully understood manner [41]. Similar to some other species within the *Plumbaginaceae* family, *A. maritima* possesses salt glands on the surface of its leaves (Figure 1b) [41,42]. Despite extensive research on the physiology and adaptation mechanisms of this species, the nature and formation process of its crystalline deposits remain poorly understood. These specialised secretory structures primarily serve to expel salt solutes from tissues when the soil’s salt concentration exceeds the plant’s tolerance levels [43]. This adaptation is typical for halophytes—plants that grow in saline soils [44]. Importantly, the multi-ionic solutes excreted onto the leaf surface undergo crystallisation and form conglomerates of minuscule crystals (Figure 1b) [17]. We selected this particular plant due to our involvement in a broader project focused on environmental aspects, namely its tolerance for heavy metal pollution. In our previous study, we investigated the chemical composition and some structural features of *Armeria*-derived bio-crystals using X-ray crystallography and TEM microscopy. Crystallographic studies allowed us to identify several inorganic salts. Some of them, including potassium calcium sulphate monohydrate and potassium magnesium sulphate hexahydrate, have not been reported in the context of biological crystallisation processes [17]. This work is a follow-up to the previously mentioned study and is focused more on the methodological aspects of studies on biomineralisation. Here, for the first time, microED has been applied to plant-derived crystals, offering a novel approach to understanding biomineralisation in plants. Our research reveals the presence of sodium chloride (halite), sodium sulphate (thénardite), and calcium sulphate dihydrate (gypsum) in very small crystalline conglomerates, which would be virtually impossible or very tedious to analyse using traditional X-ray crystallography. These findings highlight the potential of microED to uncover unique structural features of biological crystals, ultimately contributing to a more comprehensive understanding of the plant’s adaptation mechanisms to harsh environments.

## 2. Results

For the current study, *A. maritima* plants were germinated using seeds collected from plants growing on a zinc-lead heap in Bolesław (Lesser Poland Voivodeship, Poland). This site, located in close proximity to a non-ferrous metal smelter, is enriched with heavy metals, including Pb, Zn, Cd, and Tl. After germination, plants were cultivated for six months under laboratory conditions using a growth medium enriched with either cadmium (Cd) or thallium (Tl) cations. Upon completion, mature leaves from each plant were collected to examine the crystals deposited on their surface. Representative images of these crystal clusters, taken using a scanning electron microscope, are provided in the Supplementary Material (Figure S1). It should be noted that the plant samples utilised in this study came from a more extensive project, which was mentioned earlier in the introduction to this article [17]. The rationale for selecting cadmium- and thallium-exposed plants stemmed from the subpar quality and diminutive size of their excreted crystalline materials, rendering them unsuitable for routine X-ray crystallography but ideal for microED analysis.

**Figure 2:**
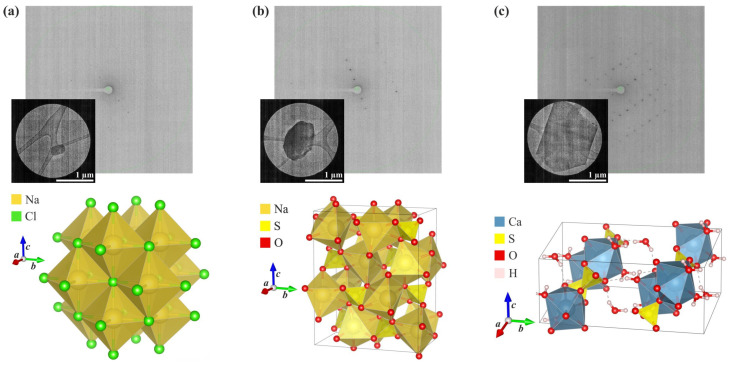
Results of the microED analysis of crystals from the surface of *A. maritima* leaves (the measured microcrystal, an exemplary frame showing the diffraction signal, and the crystal packing of the compound): (**a**) sodium chloride (halite), (**b**) sodium sulphate (thénardite), and (**c**) calcium sulphate dihydrate (gypsum).

In the next step, crystalline material taken from the surface of the leaves was placed on glow-discharged lace carbon 200 mesh Cu grids and exposed to a low-dose electron beam to record diffraction patterns using a Glacios cryo-transmission electron microscope (cryo-TEM) with a field emission gun. Collected diffraction images were further processed using generally available crystallographic programmes including the XDS and SHELX packages. Importantly, the small size and overall suboptimal quality of the plant-derived crystals did not hinder microED’s ability to determine the chemical composition and structure of the analysed samples. As a result, the crystal structures of three inorganic salts were established: sodium chloride, sodium sulphate, and calcium sulphate dihydrate (Figure 2).

**Figure 3:**
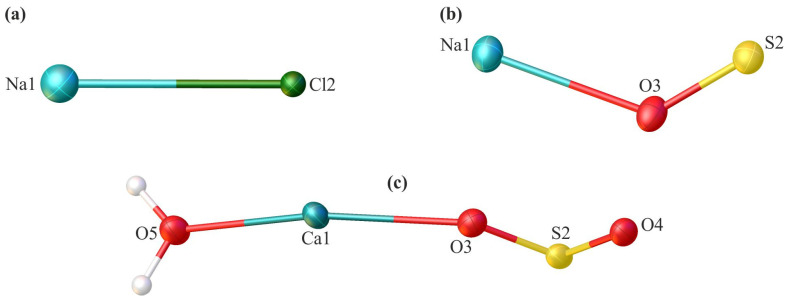
The asymmetric unit of the crystal lattice of the investigated compounds—sodium chloride (**a**), sodium sulphate (**b**), and calcium sulphate dehydrate (**c**)—with the atom labelling scheme. Displacement ellipsoids are drawn at the 50% probability level.

The first two systems were derived from plants exposed to cadmium, and the last one was from a thallium-treated plant. The content of the asymmetric unit of the crystal lattice for each investigated compound is illustrated in Figure 3. Comprehensive crystallographic data, along with the refinement parameters, are presented in Table 1. An exhaustive list of bond lengths and valence and torsion angles is available in Supplementary Tables S1–S8. The structural features of all inorganic salts from the analysed set align well with the minerals whose structures were determined previously using X-ray diffraction. The sodium chloride had the structure of halite (cubic, space group *Fm*-*3m*) [45,46], anhydrous sodium sulphate was identified as thénardite (orthorhombic, space group *Fddd*) [47], and the sodium sulphate dihydrate was consistent with gypsum (monoclinic, space group *C*2/*c*) [48].

The quality of the collected ED data is typical for microED methods applied to imperfect crystalline samples. High values of *R*-factors and other figures of merit arise not necessarily from defects in the crystal structure or instrumental effects, but largely from dynamical diffraction effects caused by multiple scatterings of electrons within the crystal lattice. Given that electrons interact more robustly with matter than photons, these secondary effects cannot be disregarded as they are often in X-ray crystallography [49]. Regrettably, the methods allowing for the dynamical refinement of ED data are still in their infancy and currently apply only to very high-quality samples [50].

## 3. Discussion

While the crystal structures of all examined compounds have been described previously, our findings are valuable from the perspective of plant-induced crystallisation. Sodium chloride is an expected product of the salt gland of *A. maritima*, but to our knowledge, its structure has never been solved using microED. Although it is known that SO_4_^2−^ and Ca^2+^ ions are secreted by salt glands, the discovery that gypsum can form bio-induced crystals is very recent [17], and this phenomenon is unique to *A. maritima*. Given the size of the crystals we measured (Figure 2c), it is noteworthy that the quality of our ED data enabled us to pinpoint the correct positions of hydrogen atoms without using geometrical approximations during model refinement [51]. This level of precision is challenging to achieve with X-ray diffraction applied to environmental samples. Traditional methods of X-ray refinement lead to shortened H–X bond lengths (where X is a non-hydrogen atom), while more advanced techniques, including HAR (Hirshfeld Atom Refinement), demand high-quality crystals [52]. In gypsum, water molecules form robust hydrogen bonds with sulphate anions.

**Figure 4:**
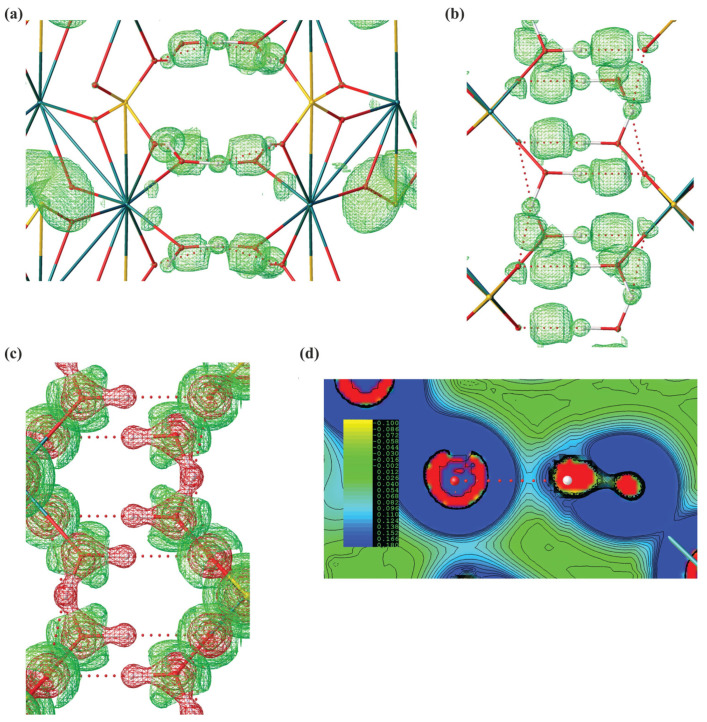
Computational analysis of CaSO_4_ 2H_2_O system. (**a**) Analysis of hydrogen bonds with isosurfaces of ELI-D (2.7) along *x*-90° and *z*-90° axis. (**b**) Large basins of ELI-D indicate the regions in which the likelihood of finding an electron pair relative to the whole molecular system is high. A visible basin of ELI-D along a hydrogen bond indicates a significant covalent contribution. ELI-D is a dimensionless quantity. (**c**) Isosurfaces of ED Laplacian along x-90° axis (0.5 e A^−5^). (**d**) Contour map of ED Laplacian along a hydrogen bond.

Leveraging our experimental structure, we successfully computed electron density Laplacian and ELI-D (Electron Localizability Index) for the obtained experimental structure of gypsum, revealing a pronounced covalent component in these bonds (Figure 4). Such covalent contribution is often found in ionic hydrogen bonds, including inorganic systems such as hydrated ionic clusters. Intriguingly, the anhydrous sodium sulphate, consistent with the structure of thénardite, has never been mentioned in the context of biomineralisation or bio-induced crystallisation processes, whether in plants or other organisms. The emergence of this salt in its anhydrous state is somewhat unexpected due to its hygroscopic nature. In the presence of moisture, thénardite gradually transitions into its hydrated form (Na_2_SO_4_·H_2_O; mineral mirabilite) [53]. We speculate that thénardite crystals on the *A. maritima* leaves might be covered by a protective film, possibly composed of nanoparticle gypsum or another substance, which inhibits or significantly lowers the rate of its hydration. There is some indirect evidence that oxalate crystals present in some plants may be at least partially covered by a layer of biological materials such as membranes or polysaccharides.

Considering the detection of these compounds, our results align with prior studies indicating that *A. maritima* can efficiently remove excessive sulphate ions [17]. Elevated cellular sulphate levels often have a detrimental effect on plant physiology, making the regulation of their uptake and internal distribution critical for proper metabolism, especially in combination with exposure to high levels of heavy metal cations [54,55]. *A. maritima*, which is capable of transforming this anion into practically insoluble gypsum, could be considered a promising candidate for the recultivation of sulphate-contaminated soils.

In conclusion, our study highlights the efficacy of microED as a valuable tool for investigating biomineralisation in plants, even when dealing with minimal and imperfect crystal samples. The ability of microED to provide detailed structural insights from such challenging materials underscores its potential for broader application in plant science. As numerous plant species are known to produce diverse crystalline materials, with many more yet to be explored, microED offers a promising avenue for advancing our understanding of these processes. The increasing accessibility of this technique, coupled with ongoing advancements in user-friendly software for data processing and structure refinement, further strengthens its appeal. Given these advancements, we firmly advocate for the wider adoption of microED within the scientific community. When you are faced with a dilemma—“to have a crystal structure or not”—do not hesitate to consider using microED to solve various crystallographic challenges.

## 4. Materials and Methods

### 4.1. Cultivation of A. maritima Plants

The *A. maritima* plants were germinated using seeds collected from plants growing on a zinc-lead heap in Bolesław (Lesser Poland Voivodeship, Poland). The seeds were germinated on a damp filter paper in Petri dishes at 15 °C. After germination, seedlings were relocated to a greenhouse and planted in containers filled with perlite. The growth medium was ½-diluted Knop’s medium. Cadmium (Cd) or thallium (Tl) was added to the medium in the form of nitrate at a concentration of 1 mg/L and 0.5 mg/L, respectively. This heavy metal-enriched medium was refreshed biweekly. The cultivation period spanned six months, covering the full developmental cycle of *A. maritima*. After this time, mature leaves from each plant were collected to examine the crystals deposited on their surface.

### 4.2. SEM Visualisation of Crystalline Material

The images visualising the crystalline material on the dried mature leaves were recorded using a Phenom ProX scanning electron microscope (SEM) (Thermo-Fisher, Waltham, MA, USA).

### 4.3. MicroED Measurements

Crystalline material was carefully scraped from the leaf surface using a steel needle, deposited onto a Petri dish, and broken down into smaller particles using a steel spatula. The prepared samples were then placed on glow-discharged lace carbon 200-mesh Cu grids and exposed to a low-dose electron beam to record diffraction patterns. Data collection was performed using a Glacios cryo-transmission electron microscope (cryo-TEM) with a field emission gun, operating at 200 kV and −192 °C (Thermo-Fisher, Waltham, MA, USA). The microscope was equipped with the CETA-D detector (Thermo-Fisher) and an autoloader with a twelve-grid holder. Configuration settings included a 50 μm condenser aperture, spot size 11, and gun lens 8. Diffraction datasets were gathered under parallel illumination conditions set up at a magnification of 28kx (physical pixel size 0.52 nm) and at an exceptionally low dose (mean dose rate of 0.0285 e/Å2/s, total dose of 3.4 e/A2). The crystal was methodically rotated between −60° and +60° with a tilt angle of 0.5° and a tilt speed of 1° per second. While the microscope operated in diffraction mode with a calibrated camera length (CL) of 652 mm, the camera continuously captured data in rolling shutter mode. Settings for this mode included hardware binning 2 (which corresponds to the detector pixel size of 28 µm) and an exposure time of 0.5 s. Data were collected using EPU-D 1.14 software (Thermo-Fisher) and saved in the SMV format with metadata for further processing in standard crystallographic programmes.

### 4.4. Determination of the Crystal Structures and Computational Studies

Diffraction data were reduced using the XDS programme [56]; both the crystal structure solution and the refinement were carried out using the SHELX 2018/3 package [57]. Images of crystal structures were prepared in the VESTA 3 programme [58]. Molecular wavefunctions were necessary for the calculations of ED Laplacian and ELI-D, which were computed in the ORCA 5.0 package [59,60] at the wB97X/def2-TZVPD level of theory [61,62] using the TRAH-SCF procedure [63]. The ED Laplacian and ELI-D grids were calculated in NoSpherA2 program [64] via Olex2.15 software [65]. All maps were visualised and rendered in Olex2.15 [65].

## Figures and Tables

**Table 1:** Crystal data and structure refinement details for investigated compounds.

Compound	Sodium Chloride	Sodium Sulphate	Calcium Sulphate Dihydrate
Empirical formula	NaCl	Na_2_SO_4_	CaH_4_O_6_S
Formula weight	58.44	142.04	172.17
Temperature/K	80	80	80
Crystal system	cubic	orthorhombic	monoclinic
Space group	*Fm*-3*m*	*Fddd*	*C*2/*c*
*a*/Å	5.50(10)	5.600(10)	6.10(10)
*b*/Å	5.50(10)	9.600(10)	14.70(10)
*c*/Å	5.50(10)	12.000(10)	5.60(10)
*α*/°	90	90	90
*β*/°	90	90	114.30
*γ*/°	90	90	90
Volume/Å^3^	166(9)	645.1(14)	458(12)
*Z*	4	8	4
*ρ*_calc_g/cm^3^	2.333	2.925	2.499
*μ*/mm^−1^	0.000	0.000	0.000
*F*(000)	38.0	181.0	115.0
Radiation	*λ* = 0.0251 Å	*λ* = 0.0251 Å	*λ* = 0.0251 Å
2*Θ* range for data collection/°	0.452 to 1.734	0.32 to 2.542	0.31 to 2.526
Index ranges	−5 ≤ *h* ≤ 5, −6 ≤ *k* ≤ 6, −6 ≤ *l* ≤ 6	−9 ≤ *h* ≤ 9, −16 ≤ *k* ≤ 16, −19 ≤ *l* ≤ 19	−10 ≤ *h* ≤ 10, −25 ≤ *k* ≤ 25, −9 ≤ *l* ≤ 9
Reflections collected	219	2194	3084
Independent reflections	21 [*R*_int_ = 0.3562, *R*_sigma_ = 0.2103]	428 [*R*_int_ = 0.2203, *R*_sigma_ = 0.1617]	995 [*R*_int_ = 0.2357, *R*_sigma_ = 0.2551]
Data/restraints/parameters	21/0/4	428/0/19	995/2/47
Goodness-of-fit on *F*^2^	1.597	1.294	1.117
Final *R* indices [*I* ≥ 2σ (*I*)]	*R*_1_ = 0.1341, w*R*_2_ = 0.3094	*R*_1_ = 0.1247, w*R*_2_ = 0.3762	*R*_1_ = 0.1510, w*R*_2_ = 0.3920
Final *R* indices [all data]	*R*_1_ = 0.2009, w*R*_2_ = 0.4069	*R*_1_ = 0.1634, w*R*_2_ = 0.4066	*R*_1_ = 0.2418, w*R*_2_ = 0.4551
Largest diff. peak/hole/Å^−2^	0.20/−0.41	0.27/−0.19	0.28/−0.22

## Data Availability

CSD 2351881–2351883 contain the supplementary crystallographic data for this paper. These data can be obtained free of charge from FIZ Karlsruhe via www.ccdc.cam.ac.uk/structures, accessed on 14 October 2024.

## Supplementary Materials

The following supporting information can be downloaded at https://www.mdpi.com/article/10.3390/molecules29204916/s1.
