# Supplementary material for: MicroED: Unveiling the Structural Chemistry of Plant Biomineralisation
Source: Molecules. 2024 Oct 17;29(20):4916. doi: 10.3390/molecules29204916 (PMC11510860; doi:10.3390/molecules29204916)
Supplement: Supplementary file 1 [file molecules-29-04916-s001.zip › molecules-3252488-supplementary.pdf]

Electronic Supplementary Information (ESI)

MicroED: Unveiling the Structural Chemistry of Plant Biomineralisation

Damian Trzybiński [1,†], Marcin Ziemniak [1,†], Barbara Olech [1,2], Szymon Sutuła [1,2], Tomasz Góral [2], Olga Bemowska-Kałabun [3], Krzysztof Brzost [4], Małgorzata Wierzbicka [4,*] and Krzysztof Woźniak [1,*]

[1] Faculty of Chemistry, Biological and Chemical Research Centre, University of Warsaw, Żwirki i Wigury 101, 02-089 Warsaw, Poland; dtrzybinski@cnbc.uw.edu.pl (D.T.); mziemniak@chem.uw.edu.pl (M.Z.); b.gruza@uw.edu.pl (B.O.); s.sutula@uw.edu.pl (S.S.)

[2] Centre of New Technologies, University of Warsaw, Banacha 2C, 02-097 Warsaw, Poland; t.goral@cent.uw.edu.pl

[3] Isotope Laboratory, Faculty's Independent Centres, Faculty of Biology, University of Warsaw, Miecznikowa 1, 02-096 Warsaw, Poland; o.bemowska@uw.edu.pl

[4] Department of Ecotoxicology, Institute of Environmental Biology, Faculty of Biology, University of Warsaw, Miecznikowa 1, 02-096 Warsaw, Poland; brzost@biol.uw.edu.pl

* Correspondence: wierzbicka@biol.uw.edu.pl (M.W.); kwozniak@chem.uw.edu.pl (K.W.)

† These authors contributed equally to this work.

a)

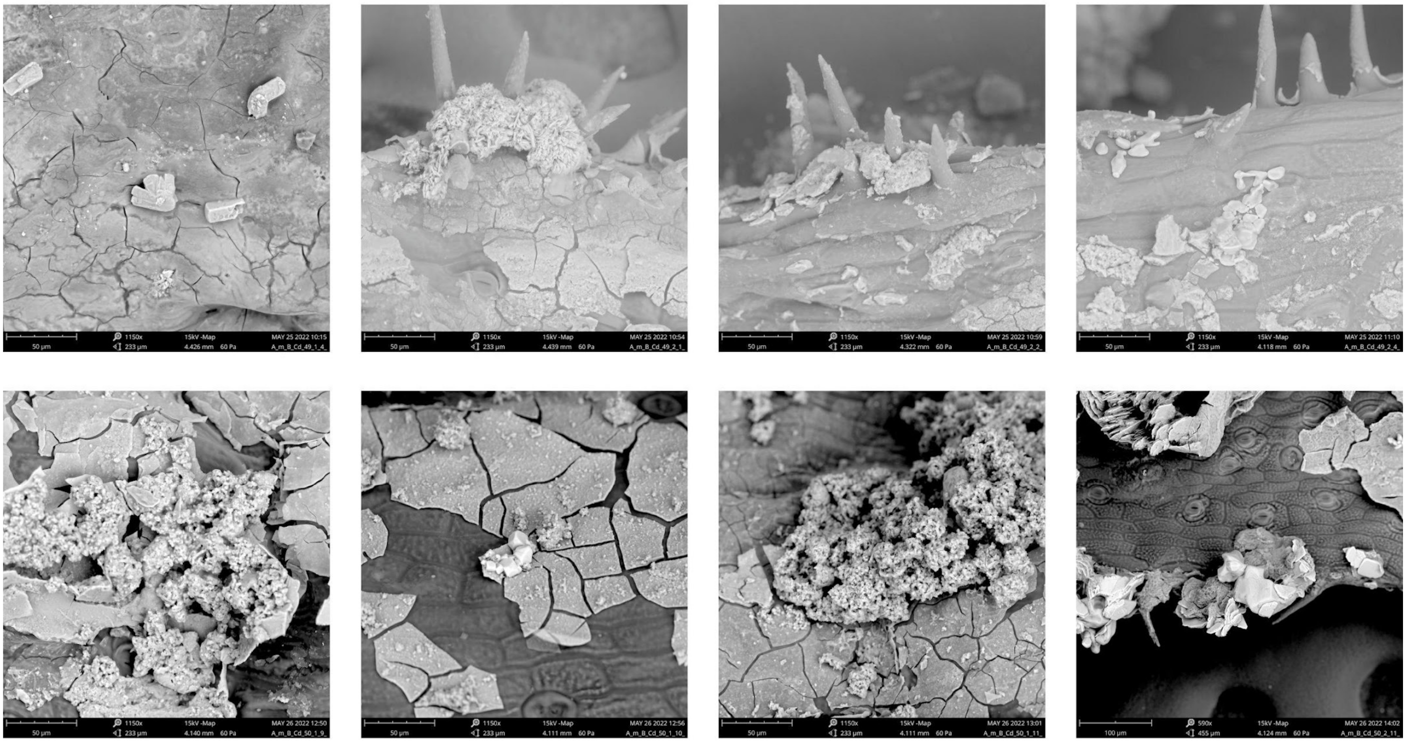

b)

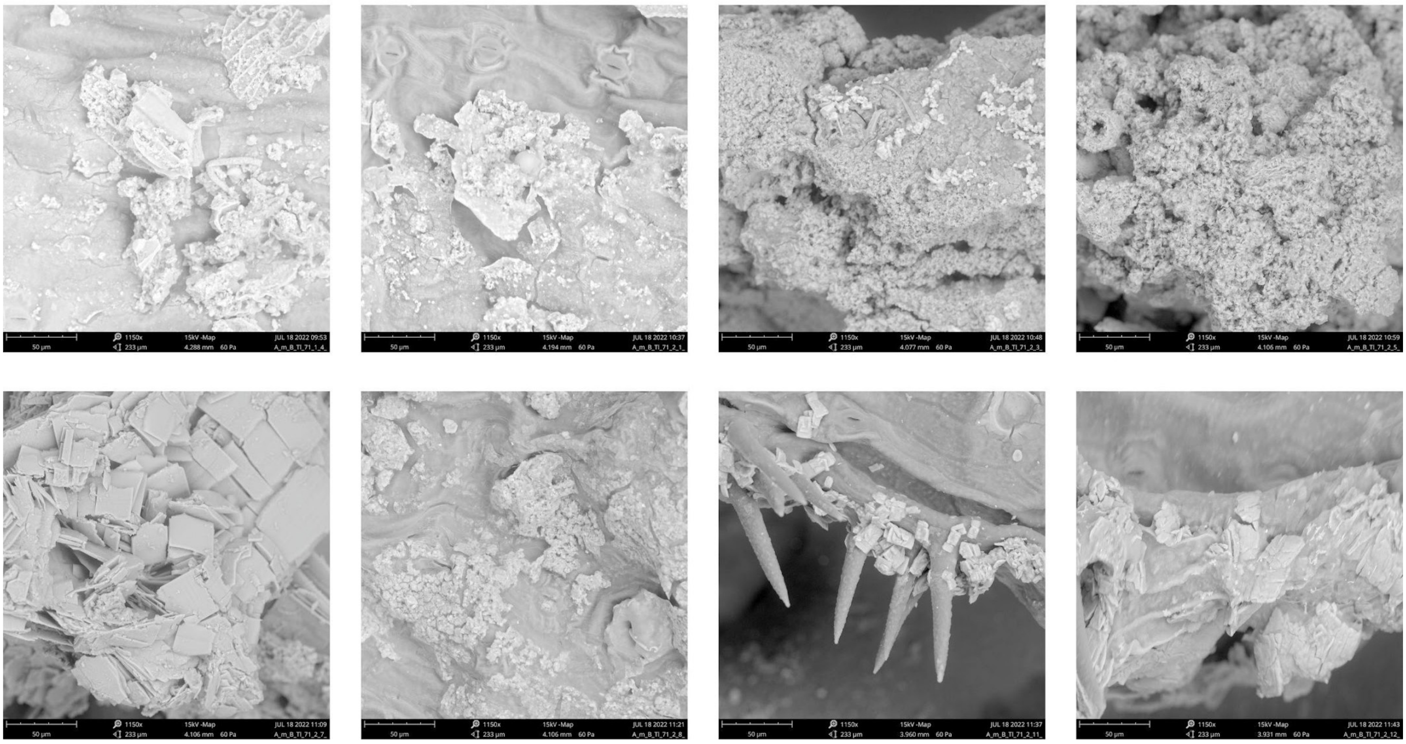

Figure S1. Examples of crystalline material formed on the surface of the leaves of *A. maritima* plants after exposure to cadmium (d) and thallium

Table S1. Bond lengths for investigated sodium chloride.

Atom	Atom	Length/Å	Atom	Atom	Length/Å
Cl01	Na02^{1}	2.75(5)	Cl01	Na02^{3}	2.75(5)
Cl01	Na02^{2}	2.75(5)	Cl01	Na02^{4}	2.75(5)
Cl01	Na02	2.75(5)	Cl01	Na02^{5}	2.75(5)

^{1}1/2+X,1/2+Y,+Z; ^{2}1/2+X,-1/2+Y,+Z; ^{3}1/2+X,+Y,1/2+Z; ^{4}1+X,+Y,+Z; ^{5}1/2+X,+Y,-1/2+Z

Table S2. Valence angles for investigated sodium chloride.

Atom	Atom	Atom	Angle/°	Atom	Atom	Atom	Angle/°
Na02^{1}	Cl01	Na02	90.0	Cl01^{6}	Na02	Cl01	90.0
Na02^{2}	Cl01	Na02^{3}	180.0	Cl01^{7}	Na02	Cl01^{8}	180.0
Na02^{2}	Cl01	Na02^{4}	90.0	Cl01^{7}	Na02	Cl01^{9}	90.0
Na02^{5}	Cl01	Na02^{3}	90.0	Cl01^{10}	Na02	Cl01^{8}	90.0
Na02	Cl01	Na02^{4}	90.0	Cl01	Na02	Cl01^{9}	90.0
Na02^{5}	Cl01	Na02^{4}	90.0	Cl01^{10}	Na02	Cl01^{9}	90.0
Na02^{1}	Cl01	Na02^{2}	90.0	Cl01	Na02	Cl01^{8}	90.0
Na02^{1}	Cl01	Na02^{5}	90.0	Cl01^{6}	Na02	Cl01^{10}	90.0
Na02	Cl01	Na02^{2}	90.0	Cl01	Na02	Cl01^{7}	90.0
Na02^{1}	Cl01	Na02^{4}	180.0	Cl01^{6}	Na02	Cl01^{9}	180.0
Na02^{5}	Cl01	Na02^{2}	90.0	Cl01^{10}	Na02	Cl01^{7}	90.0
Na02	Cl01	Na02^{5}	180.0	Cl01	Na02	Cl01^{10}	180.0
Na02^{1}	Cl01	Na02^{3}	90.0	Cl01^{6}	Na02	Cl01^{8}	90.0
Na02	Cl01	Na02^{3}	90.0	Cl01^{8}	Na02	Cl01^{9}	90.0
Na02^{3}	Cl01	Na02^{4}	90.0	Cl01^{6}	Na02	Cl01^{7}	90.0

^{1}1/2+X,1/2+Y,+Z; ^{2}1/2+X,+Y,-1/2+Z; ^{3}1/2+X,+Y,1/2+Z; ^{4}1/2+X,-1/2+Y,+Z; ^{5}1+X,+Y,+Z; ^{6}-1/2+X,1/2+Y,+Z; ^{7}-1/2+X,+Y,-1/2+Z; ^{8}-1/2+X,+Y,1/2+Z; ^{9}-1/2+X,-1/2+Y,+Z; ^{10}-1+X,+Y,+Z

Table S3. Bond lengths for investigated sodium sulfate.

Atom	Atom	Length/Å	Atom	Atom	Length/Å
S2	O3	1.441(4)	Na1	O3^{5}	2.329(5)
S2	O3^{1}	1.441(4)	Na1	O3^{6}	2.265(4)
S2	O3^{2}	1.441(4)	Na1	O3^{7}	2.474(6)
S2	O3^{3}	1.441(4)	Na1	O3^{8}	2.329(5)
Na1	Na1^{3}	3.082(6)	Na1	O3^{9}	2.474(6)
Na1	Na1^{4}	3.082(6)	Na1	O3	2.265(4)

^{1}1/4-X,+Y,5/4-Z; ^{2}+X,5/4-Y,5/4-Z; ^{3}1/4-X,5/4-Y,+Z; ^{4}5/4-X,5/4-Y,+Z; ^{5}1-X,3/2-Y,3/2-Z; ^{6}3/4-X,+Y,7/4-Z; ^{7}1/4+X,-1/4+Y,3/2-Z; ^{8}-1/4+X,3/2-Y,1/4+Z; ^{9}1/2-X,-1/4+Y,1/4+Z

Table S4. Valence angles for investigated sodium chloride.

Atom	Atom	Atom	Angle/°	Atom	Atom	Atom	Angle/°
O3	S2	O3^{1}	111.1(3)	O3^{8}	Na1	O3	169.2(3)
O3^{1}	S2	O3^{2}	110.4(3)	O3	Na1	O3^{5}	108.53(19)
O3	S2	O3^{2}	106.9(3)	O3	Na1	O3^{7}	81.06(15)
O3	S2	O3^{3}	110.4(3)	O3	Na1	O3^{6}	81.31(12)
O3^{3}	S2	O3^{2}	111.1(3)	O3^{8}	Na1	O3^{9}	81.06(15)
O3^{1}	S2	O3^{3}	106.9(3)	O3^{7}	Na1	O3^{5}	137.63(17)
Na1^{3}	Na1	Na1^{4}	130.6(3)	O3	Na1	O3^{9}	94.74(14)
O3	Na1	Na1^{4}	105.92(10)	O3^{8}	Na1	O3^{7}	94.74(14)
O3^{5}	Na1	Na1^{4}	86.0(2)	O3^{7}	Na1	O3^{9}	134.7(3)
O3^{6}	Na1	Na1^{3}	86.0(2)	O3^{7}	Na1	O3^{6}	86.71(16)
O3^{7}	Na1	Na1^{4}	52.17(12)	O3^{8}	Na1	O3^{5}	81.31(12)
O3^{5}	Na1	Na1^{3}	48.04(14)	O3^{9}	Na1	O3^{5}	86.71(16)
O3	Na1	Na1^{3}	78.73(9)	O3^{8}	Na1	O3^{6}	108.53(19)
O3^{8}	Na1	Na1^{4}	78.73(9)	O3^{9}	Na1	O3^{6}	137.63(17)

O3^{7}	Na1	Na1^{3}	159.32(11)	S2	O3	Na1	129.7(2)
O3^{9}	Na1	Na1^{3}	52.17(13)	S2	O3	Na1^{7}	120.7(2)
O3^{9}	Na1	Na1^{4}	159.32(11)	S2	O3	Na1^{10}	98.65(19)
O3^{8}	Na1	Na1^{3}	105.92(10)	Na1	O3	Na1^{10}	119.2(2)
O3^{6}	Na1	Na1^{4}	48.04(14)	Na1^{7}	O3	Na1^{10}	79.79(14)
O3^{5}	Na1	O3^{6}	55.8(2)	Na1	O3	Na1^{7}	98.94(15)

^{1}+X,5/4-Y,5/4-Z; ^{2}1/4-X,+Y,5/4-Z; ^{3}1/4-X,5/4-Y,+Z; ^{4}5/4-X,5/4-Y,+Z; ^{5}1/2-X,-1/4+Y,1/4+Z; ^{6}1/4+X,-1/4+Y,3/2-Z; ^{7}1-X,3/2-Y,3/2-Z; ^{8}3/4-X,+Y,7/4-Z; ^{9}-1/4+X,3/2-Y,1/4+Z; ^{10}-1/4+X,1/4+Y,3/2-Z

Table S5. Torsion angles for investigated sodium sulfate.

A	B	C	D	Angle/°	A	B	C	D	Angle/°
O3^{1}	S2	O3	Na1	-99.3(3)	O3^{2}	S2	O3	Na1	140.2(3)
O3^{2}	S2	O3	Na1^{3}	0.000(1)	O3^{4}	S2	O3	Na1^{3}	-121.01(18)
O3^{1}	S2	O3	Na1^{3}	120.54(19)	O3^{1}	S2	O3	Na1^{5}	37.33(15)
O3^{4}	S2	O3	Na1	19.15(16)	O3^{4}	S2	O3	Na1^{5}	155.8(3)
O3^{2}	S2	O3	Na1^{5}	-83.2(2)					

^{1}+X,5/4-Y,5/4-Z; ^{2}1/4-X,+Y,5/4-Z; ^{3}-1/4+X,1/4+Y,3/2-Z; ^{4}1/4-X,5/4-Y,+Z; ^{5}1-X,3/2-Y,3/2-Z

Table S6. Bond lengths for investigated calcium sulfate dihydrate.

Atom	Atom	Length/Å	Atom	Atom	Length/Å
Ca1	O5	2.31(2)	Ca1	O4^{2}	2.46(3)
Ca1	O5^{1}	2.31(2)	Ca1	O4^{3}	2.46(3)
Ca1	O3^{2}	2.46(4)	S2	O3	1.448(15)
Ca1	O3	2.32(3)	S2	O3^{4}	1.448(15)
Ca1	O3^{1}	2.32(3)	S2	O4^{4}	1.448(15)
Ca1	O3^{3}	2.46(4)	S2	O4	1.448(15)

^{1}1-X,+Y,1/2-Z; ^{2}-1/2+X,3/2-Y,-1/2+Z; ^{3}3/2-X,3/2-Y,1-Z; ^{4}1-X,+Y,3/2-Z

Table S7. Valence angles for investigated calcium sulfate dihydrate.

Atom	Atom	Atom	Angle/°	Atom	Atom	Atom	Angle/°
O5	Ca1	O5^{1}	99.3(9)	O3	Ca1	O4^{2}	124.0(7)
O5^{1}	Ca1	O3	84.1(11)	O3	Ca1	O4^{3}	80.6(8)
O5	Ca1	O3^{2}	131.8(3)	O3^{1}	Ca1	O4^{3}	124.0(7)
O5	Ca1	O3^{1}	84.1(11)	O3^{1}	Ca1	O4^{2}	80.6(8)
O5	Ca1	O3^{3}	77.7(6)	O4^{3}	Ca1	O3^{3}	55.9(9)
O5^{1}	Ca1	O3^{1}	160.0(4)	O4^{3}	Ca1	O3^{2}	140.9(8)
O5^{1}	Ca1	O3^{2}	77.7(6)	O4^{2}	Ca1	O3^{3}	140.9(8)
O5	Ca1	O3	160.0(4)	O4^{2}	Ca1	O3^{2}	55.9(9)
O5^{1}	Ca1	O3^{3}	131.8(3)	O4^{3}	Ca1	O4^{2}	144.3(6)
O5^{1}	Ca1	O4^{2}	81.1(11)	O3	S2	O3^{4}	111.1(9)
O5^{1}	Ca1	O4^{3}	76.0(11)	O3	S2	O4	105.6(11)
O5	Ca1	O4^{3}	81.1(11)	O3^{4}	S2	O4^{4}	105.6(11)
O5	Ca1	O4^{2}	76.0(11)	O3^{4}	S2	O4	110.8(11)
O3^{1}	Ca1	O3^{2}	85.3(4)	O3	S2	O4^{4}	110.8(11)
O3^{1}	Ca1	O3^{3}	68.2(4)	O4^{4}	S2	O4	113.1(9)
O3^{1}	Ca1	O3	99.4(12)	Ca1	O3	Ca1^{2}	111.8(4)
O3^{2}	Ca1	O3^{3}	139.0(7)	S2	O3	Ca1^{2}	99.1(9)
O3	Ca1	O3^{3}	85.3(4)	S2	O3	Ca1	148.6(7)
O3	Ca1	O3^{2}	68.2(4)	S2	O4	Ca1^{2}	99.4(12)

^{1}1-X,+Y,1/2-Z; ^{2}3/2-X,3/2-Y,1-Z; ^{3}-1/2+X,3/2-Y,-1/2+Z; ^{4}1-X,+Y,3/2-Z

Table S8. Torsion angles for investigated calcium sulfate dihydrate.

A	B	C	D	Angle/°	A	B	C	D	Angle/°
O3^{1}	S2	O3	Ca1	67.6(9)	O4	S2	O3	Ca1	-172.3(6)

O3[1]	S2	O3	Ca1[2]	-122.7(6)	O4	S2	O3	Ca1[2]	-2.5(3)
O3	S2	O4	Ca1[2]	2.6(3)	O4[1]	S2	O3	Ca1[2]	120.3(6)
O3[1]	S2	O4	Ca1[2]	123.0(7)	O4[1]	S2	O4	Ca1[2]	-118.7(8)
O4[1]	S2	O3	Ca1	-49.5(9)					

[1]1-X,+Y,3/2-Z; [2]3/2-X,3/2-Y,1-Z
